# Adapted motivational interviewing to improve the uptake of treatment for glaucoma in Nigeria: study protocol for a randomised controlled trial

**Published:** 2014

**Authors:** Mohammed M Abdull, Clare Gilbert, Jim McCambridge, Jennifer Evans

**Mohammed M Abdull, Clare Gilbert, Jim McCambridge** and **Jennifer Evans**

Trials 2014, 15:149 doi:10.1186/1745-6215-15-149

## Background

Glaucoma is a chronic eye disease associated with irreversible visual loss. In Africa, glaucoma patients often present late, with very advanced disease. One-off procedures, such as laser or surgery, are recommended in Africa because of lack of or poor adherence to medical treatment. However, acceptance of surgery is usually extremely low. To prevent blindness, adherence to treatment needs to improve, using acceptable, replicable and cost-effective interventions. After reviewing the literature and interviewing patients in Bauchi (Nigeria) motivational interviewing (MI) was selected as the intervention for this trial, with adaptation for glaucoma (MIG). MI is designed to strengthen personal motivation for, and commitment to a specific goal by eliciting and exploring a person's reasons for change within an atmosphere of acceptance and compassion. The aim of this study is to assess whether MIG increases the uptake of laser or surgery amongst glaucoma patients where this is the recommended treatment. The hypothesis is that MIG increases the uptake of treatment. This will be the first trial of MI in Africa.

## Methods

This is a hospital-based, single-centre, randomised controlled trial of MIG plus an information sheet on glaucoma and its treatment (the latter being ‘standard care’) compared with standard care alone for glaucoma patients where the treatment recommended is surgery or laser.

Those eligible for the trial are adults aged 17 years and above who live within 200 km of Bauchi with advanced glaucoma where the examining ophthalmologist recommends surgery or laser.

After obtaining written informed consent, participants will be randomly allocated to MIG plus standard care, or standard care alone. Motivational interviewing will be delivered in Hausa or English by one of two MIG trained personnel. One hundred and fifty participants will be recruited to each arm. The primary outcome is the proportion of participants undergoing laser or surgery within two months of the date given to re attend for the procedure. MIG quality will be assessed using the validated MI treatment integrity scale.

## Discussion

Motivational interviewing may be an important tool to increase the acceptance of treatment for glaucoma. The approach is potentially scalable and may be useful for other chronic conditions in Africa.

*Note:* To read more about motivational interviewing, visit **http://motivationalinterview.net/clinical/index.html**

